# Routine Troponin I Assessment Enhances Risk Stratification in Hospitalized Patients with Seasonal Influenza

**DOI:** 10.3390/jcm15124509

**Published:** 2026-06-10

**Authors:** Tobias Harm, Johannes Gernert, Monika Zdanyte, Lars Schöllmann, Karin Anne Lydia Müller, Meinrad Paul Gawaz, Dominik Rath, Simon Greulich

**Affiliations:** 1 Department of Cardiology and Angiology, University Hospital Tübingen, Eberhard Karls University Tübingen, 72076 Tübingen, Germany; 2 Department of Cardiology, Medius Clinic Ostfildern-Ruit, 73760 Ostfildern, Germany

**Keywords:** inflammation, cardiac injury, high-sensitive troponin I, influenza, machine learning, risk stratification, cardiovascular risk, coronary artery disease

## Abstract

**Background/Objectives**: Myocardial injury is linked to poor outcomes in respiratory infections. This study evaluated the prognostic value of high-sensitivity troponin I (hsTnI) in predicting 30-day outcomes in patients hospitalized with seasonal influenza. **Methods**: In this single-center retrospective study, 277 adults with laboratory-confirmed influenza were analyzed. Myocardial injury was defined by elevated hsTnI. The primary composite endpoint included 30-day mortality, intensive care unit (ICU) admission, and mechanical ventilation. **Results**: Patients with myocardial injury had significantly higher event rates for the composite endpoint than those without (*p* < 0.0001). Dynamic hsTnI elevations, reflecting acute myocardial injury, were also associated with worse outcomes (*p* = 0.026). Machine learning models incorporating hsTnI and laboratory data achieved excellent predictive performance (AUC = 0.99) and improved risk classification compared with conventional scores (*p* < 0.0001). **Conclusions**: Among hospitalized influenza patients, myocardial injury identified by hsTnI strongly predicted short-term adverse outcomes. Routine hsTnI assessment enhances risk stratification beyond standard clinical scores and may facilitate early identification and management of high-risk patients.

## 1. Introduction

Seasonal influenza remains one of the most frequent respiratory infections, leading to a high disease burden worldwide [1]. Although often a self-limiting respiratory illness, in certain cases, severe infections can lead to hospitalization, respiratory failure requiring mechanical ventilation, and even death. Historically, influenza affects nearly 10% of the global population each year, and each influenza pandemic has been associated with over 1 million deaths globally [1,2]. Moreover, the exacerbation of preexisting cardiovascular comorbidities may increase the risk of fatal outcomes in individuals with influenza infection [3,4]. Thus, influenza can lead to myocardial injury, which may manifest as myocardial infarction, myocarditis, acute heart failure, and even cardiogenic shock [3,5]. Previous studies have pointed towards an increased cardiovascular risk in patients with influenza infection, contributing to a higher incidence of cardiovascular events and increased cardiovascular mortality during the influenza season [6]. Similarly, influenza infection has been associated with an increased risk of myocardial infarction and heart failure necessitating hospitalization [5,7,8]. Therefore, influenza vaccination is recommended to attenuate the course of the infection and thereby reduce the risk of cardiovascular events in patients with cardiovascular disease [9,10,11]. During the last few years, cardiac troponin has emerged as a cornerstone biomarker for the detection of myocardial injury. We and others have previously demonstrated that elevated levels of high-sensitivity troponin I (hsTnI) are associated with increased cardiovascular risk during respiratory infections such as influenza and SARS-CoV-2 [2,12,13,14]. Importantly, elevated resting troponin concentrations may reflect not only acute myocardial injury during infection but also subclinical cardiovascular dysfunction and increased baseline cardiovascular vulnerability, even in patients without overt cardiovascular disease, which may contribute to worse prognosis during influenza infection [15,16]. However, studies assessing the role of hsTnI in infection severity and its usefulness for predicting adverse outcomes and mortality are still lacking, while contemporary risk measures often fail to precisely estimate the individual risk in patients with seasonal influenza. Therefore, the present study aims to investigate the association between elevated hsTnI serum levels and 30-day clinical outcome, including the need for mechanical ventilation, intensive care unit (ICU) admission, and all-cause mortality, in a cohort of hospitalized patients with severe influenza infection.

## 2. Materials and Methods

### 2.1. Study Population

This study was designed as an all-comers retrospective single-center cohort including consecutive adult patients hospitalized with confirmed seasonal influenza infection at the University Hospital of Tübingen, Germany, between 2016 and 2018. A total of 277 patients (*n* = 277) were included in the final analysis (Table 1). Inclusion criteria were: (1) age ≥ 18 years, (2) hospital admission during the study period, (3) laboratory-confirmed influenza infection by real-time reverse transcriptase polymerase chain reaction (RT-PCR), and (4) a documented diagnosis of influenza according to the International Statistical Classification of Diseases and Related Health Problems (ICD) [17]. Exclusion criteria were: (1) age < 18 years, (2) absence of laboratory confirmation of influenza infection, (3) missing hsTnI assessment during hospitalization, and (4) withdrawal of consent where applicable.

Seasonal influenza was identified by real-time reverse transcriptase polymerase chain reaction (RT-PCR) analysis of nasopharyngeal secretions. Patients were classified as having a confirmed influenza infection if both a diagnosis code for influenza, according to the International Statistical Classification of Diseases and Related Health Problems (ICD) [17], and laboratory confirmation of influenza were present. The severity of acute respiratory distress syndrome (ARDS) was classified based on the Berlin Definition [18]. Assessment of myocardial injury included electrocardiography (ECG), transthoracic echocardiography (TTE) findings, and measurement of hs-TnI. In line with the Fourth Universal Definition of Myocardial Infarction, myocardial injury was defined as an hs-TnI concentration above the 99th percentile [19]. Thus, using an ADVIA Centaur CP Immunoassay System (Siemens, Erlangen, Germany), the 99th percentile reference values for hs-TnI were 37 ng/L for women and 57 ng/L for men. A dynamic change in hs-TnI of ≥20% was considered clinically significant for indicating acute myocardial injury, as previously described [2,19].

All enrolled patients were admitted for the evaluation of cardiovascular disease (CVD), and invasive angiography was performed when coronary artery disease (CAD) was suspected, in accordance with current international guidelines [10,20].

In clinically stable patients, a standardized questionnaire was administered to assess medication history, cardiovascular risk factors, and comorbidities. Written informed consent was obtained from patients whenever feasible. In cases involving critically ill or deceased patients, written consent could not be obtained. The study was approved by the local ethics committee (238/2018BO2) of Tübingen. The study report followed the STROBE (Strengthening the Reporting of Observational Studies in Epidemiology) guidelines [21], and the experiments were performed in accordance with the highest ethical standards as laid down in the Declaration of Helsinki.

### 2.2. Follow-Up

Clinical follow-up for adverse events was performed over a 30-day period following hospital admission. The primary composite endpoint included all-cause mortality, the need for mechanical ventilation, and/or admission to the intensive care unit (ICU). Each individual component of the composite endpoint, including all-cause mortality, mechanical ventilation, and ICU admission, was additionally analyzed separately as a key secondary endpoint.

Follow-up data were systematically collected through comprehensive review of electronic medical records, including hospital admission records, ICU documentation, procedural reports, discharge summaries, and laboratory findings. For patients discharged before completion of the 30-day follow-up period, additional outcome information was obtained from outpatient medical records and documentation provided by general practitioners when available. Mortality status was verified using hospital records and follow-up documentation.

### 2.3. Statistical Analysis

Patient characteristics and clinical data were analyzed using JMP^®^ Pro Version 17 (SAS Institute, Cary, NC, USA) and R (R Foundation for Statistical Computing, Vienna, Austria). Normality of continuous variables was assessed using the Shapiro–Wilk test in combination with visual inspection of histograms and Q-Q plots. Normally distributed variables are presented as mean ± standard deviation (SD) and compared using the Student’s *t*-test. Non-normally distributed variables are reported as median with interquartile range (IQR) and analyzed using the Mann–Whitney U test. Categorical variables are presented as counts with percentages and compared using the chi-squared test. Correlation analysis was conducted using Pearson’s product-moment correlation coefficient and Spearman’s rank correlation coefficient, as appropriate. A comprehensive correlation matrix was generated using the “corrplot” [22] package in R. Within the “survival” [23] package in R, Cox proportional hazards models were employed to assess the association between elevated troponin levels, as well as troponin dynamics, and the 30-day composite endpoint, adjusting for age, gender, C-reactive protein, and serum creatinine levels. Cumulative hazard over time was estimated using the Nelson–Aalen estimator, and risk curves were compared across subgroups. Survival differences between subgroups were assessed using the log-rank test. For prediction of the composite endpoint, machine learning models were trained using 10-fold cross-validation. The dataset was randomly divided into a training set (90%, *n* = 249) and a test set (10%, *n* = 28). While this approach enables internal validation, the relatively small test cohort size warrants cautious interpretation of model performance estimates. We therefore compared multiple models, including extreme gradient boosting (XGBoost), support vector machines, bootstrap forest, boosted trees, neural boosted models, decision trees, nominal logistic regression, forward selection, pruned forward selection, fit stepwise, ridge, lasso, and elastic net as described previously [24,25]. Model performance was evaluated by comparing median absolute error (MAE) using the Mann–Whitney U test. Since XGBoost achieved the lowest MAE, the model was selected for final prediction and was applied to the full dataset, with automated hyperparameter tuning. Predictors with a relative gain ≥1% and all SHAP (SHapley Additive exPlanations) values were visualized using the “beeswarm” [26] package in R. Furthermore, 95% confidence intervals (CI) for the area under the receiver operating curve (ROC AUC) of XGBoost models were calculated using percentile bootstrap (10,000 repetitions) analysis. Predicted likelihoods for the composite endpoint were derived from the XGBoost formula. Final graphical outputs were generated using R and JMP.

## 3. Results

### 3.1. Baseline Characteristics, Clinical Course, and Troponin I Assessment in Influenza Patients

In the present study, we examined the association of elevated high-sensitivity troponin I (hsTnI) and the outcome of patients with seasonal influenza in a single-centre observational cohort (*n* = 277). Patients’ baseline characteristics, including medical treatment and laboratory parameters at admission, as well as concomitant diseases, including cardiovascular diseases, are summarized in Table 1.

**Figure 1:**
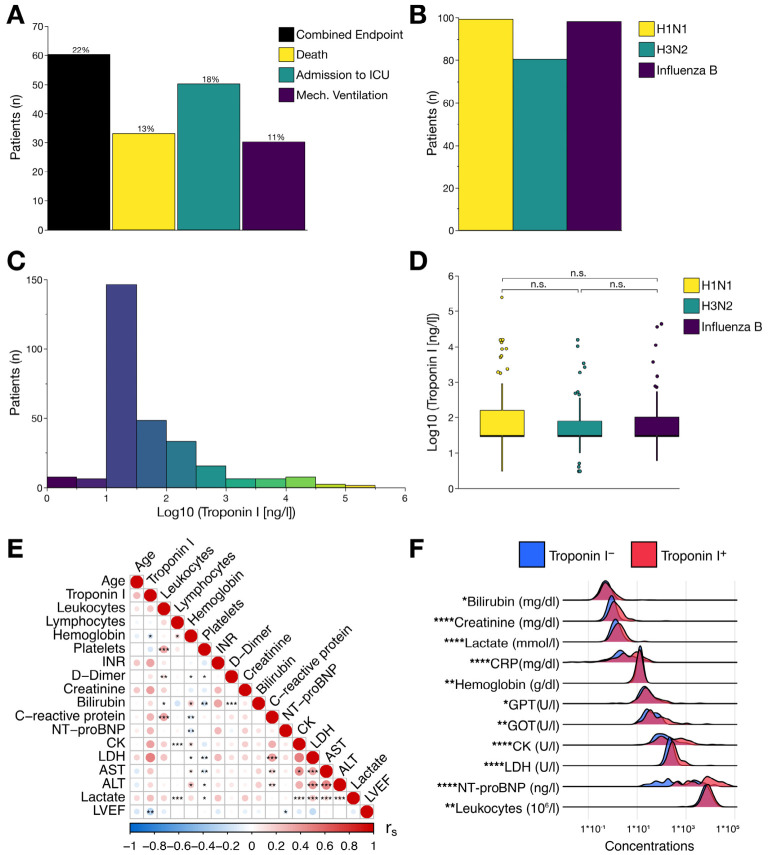
Baseline characteristics and distribution of troponin I in patients with influenza. (**A**) Incidence of the composite primary adverse endpoint (death, ICU admission, or mechanical ventilation) among influenza patients. During the 30-day follow-up period, 60 individuals (21.7%) experienced a composite primary adverse event. (**B**) Distribution of influenza A and B subtypes among the patient cohort. (**C**) Log10-transformed distribution of high-sensitivity troponin I (hsTnI) levels at admission. Histogram colors indicate increasing hsTnI concentrations, from low (blue) to high (yellow). Myocardial injury was defined as hs-TnI > 99th percentile (>37 ng/L for women and >57 ng/L for men). (**D**) Baseline hsTnI levels across did not differ among influenza virus variants (e.g., H1N1, H3N2, influenza B). (**E**) Correlation matrix displaying the association of hsTnI levels with key clinical parameters. INR, International Normalized Ratio; NT-proBNP, N-terminal pro–B-type natriuretic peptide; CK, creatine kinase; LDH, lactate dehydrogenase; AST, aspartate aminotransferase; ALT, alanine aminotransferase; LVEF, left ventricular ejection fraction. (**F**) Distribution of critical laboratory markers (e.g., lactate, inflammatory markers, creatinine, and transaminases) in patients with elevated vs. normal hsTnI. * *p* < 0.05; ** *p* < 0.01; *** *p* < 0.001; **** *p* < 0.0001.

During a 30-day follow-up period, 60 individuals (21.7%) experienced a composite primary adverse event, which included death, mechanical ventilation, or admission to the ICU (Figure 1A). Of these, 33 patients (11.9%) died, 50 individuals (18.1%) were admitted to the intensive care unit due to the severe progression of the disease, and 30 patients (10.8%) required mechanical ventilation due to severe respiratory failure (Figure 1A).

Among all patients, 179 (88.4%) were diagnosed with influenza A, and 98 (35.4%) were diagnosed with influenza B (Figure 1B). On admission, 105 patients (37.9%) had a positive hsTnI result, while 172 patients (62.1%) had a negative hsTnI result (Figure 1C). Baseline hsTnI levels did not differ significantly among patients diagnosed with different influenza variants (e.g., H1N1, H3N2, influenza B) (Figure 1D). However, we found a significant association between elevated hsTnI levels and key patient characteristics, including a strong negative correlation with left ventricular ejection fraction, hinting at myocardial impairment, as demonstrated by comprehensive correlation analysis (Figure 1E). Of note, in those patients with elevated hsTnI, we found critically enhanced laboratory values, including lactate, inflammatory parameters, creatinine, and transaminases, indicating multi-organ failure in those patients (Figure 1F).

### 3.2. Myocardial Injury Contributes to Adverse Clinical Progression in Influenza

**Figure 2:**
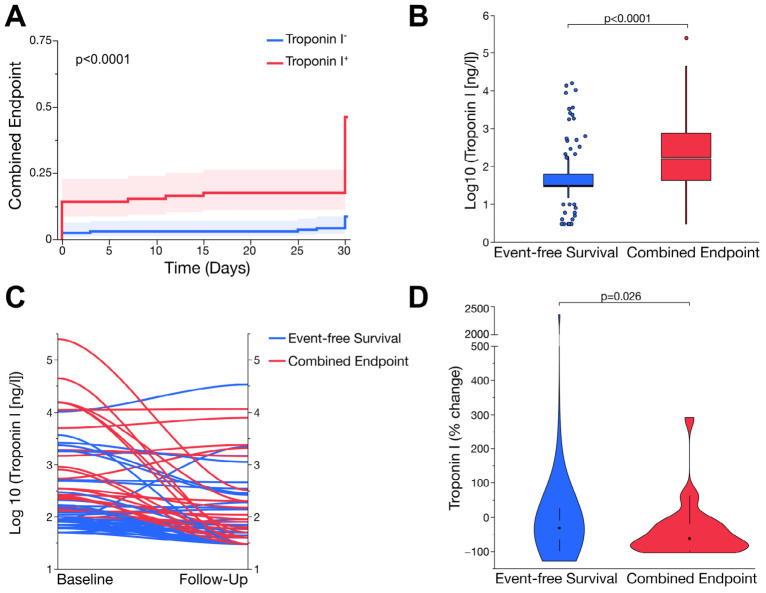
Association of hsTnI elevation with clinical outcomes in influenza patients. (**A**) Kaplan–Meier survival curves demonstrating an increased incidence of the composite endpoint in influenza patients with elevated hsTnI (*p* < 0.0001). (**B**) Quantitative analysis of hsTnI concentrations reveals increased myocardial injury in patients who experienced adverse events during follow-up. (**C**) Longitudinal analysis of hsTnI in patients with serial measurements (*n* = 68), demonstrating dynamic changes over time and hinting at acute myocardial injury (>20% change) in patients with influenza and adverse events. (**D**) A significant (*p* = 0.026) decline in hsTnI concentrations was observed among influenza patients with adverse outcomes indicating a temporal pattern of myocardial injury contributing to poor prognosis.

Therefore, we investigated the impact of elevated hsTnI levels on the clinical outcomes of patients with seasonal influenza. Kaplan–Meier survival analysis revealed a significantly (*p* < 0.0001) increased risk for the composite endpoint in patients with elevated hsTnI compared to those with normal levels (Figure 2A). Furthermore, hsTnI concentrations were directly associated with the composite outcome, as patients who experienced adverse events had significantly (*p* < 0.0001) higher hsTnI values (median 163 ng/L; IQR 42.5–757.5 ng/L) compared to those individuals without events during the follow-up (median 30 ng/L; IQR 30–60 ng/L) (Figure 2B). Furthermore, we investigated whether acute myocardial injury influences the prognosis of patients with influenza. Among patients with elevated hsTnI for whom serial measurements were available, 56 individuals (82.4%) exhibited evidence of a dynamic change in hsTnI levels over time (Figure 2C).

Interestingly, in patients who suffered from the composite endpoint, we found a significant (*p* = 0.026) drop in hsTnI levels (Figure 2D). The optimal cut-off values, determined from the Youden indices of univariable regression models and corresponding to the highest combined sensitivity and specificity, were 130 ng/L for baseline Troponin I and a 58% decrease in the repeat measurement.

### 3.3. Isolated Troponin I Elevation Is Associated with Adverse Outcomes in Influenza

**Figure 3:**
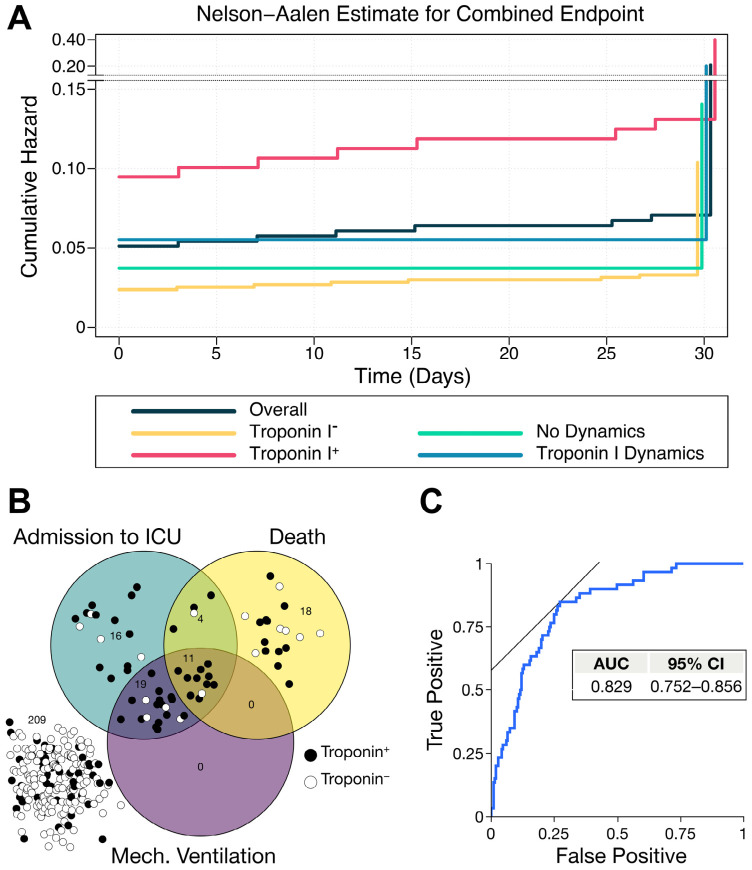
Prognostic relevance of isolated troponin I elevation in influenza. (**A**) Nelson–Aalen cumulative hazard curves for the composite endpoint in patients with elevated vs. normal hsTnI and in those with dynamic vs. stable hsTnI profiles. Indidivual Cox proportional hazards were adjusted for age, gender, creatinine, and CRP. Log-rank test indicates significant group differences (χ^2^ = 7.8, *p* < 0.05) with a critically enhanced risk of influenza patients with elevated hsTnI. (**B**) Predominant detection of elevated hsTnI among those patients who died, required mechanical ventilation, or were admitted to the ICU, supporting hsTnI was associated with specific adverse outcomes. (**C**) ROC curve of multivariable regression analysis identifying hsTnI as the only significant (*p* < 0.0001) predictor of the composite endpoint after adjustment for age, gender, influenza subtypes, cardiovascular risk factors, and comorbidities.

At 30 days following hospital discharge, the cumulative hazard of the composite endpoint was 41.4% in patients with elevated hsTnI and 10.4% in those with normal hsTnI levels (Figure 3A). In the Cox proportional hazards analysis, patients with elevated hsTnI had a significantly (*p* < 0.0001) increased risk of the composite endpoint, with an adjusted hazard ratio (HR) of 4 (95% CI 2.09–7.59), after adjustment for age, gender, creatinine, and CRP levels. Interestingly, among individuals exhibiting a dynamic change in hsTnI, suggestive of acute myocardial injury, the cumulative hazard was 21%, compared to 14.1% in patients with chronically elevated hsTnI or those with normal hsTnI levels. However, in adjusted Cox models, this difference did not reach statistical significance (HR 1.49, 95% CI 0.38–5.81; *p* = 0.568). Consequently, the adjusted Nelson–Aalen cumulative hazard curves revealed distinct divergence in event rates among the subgroups during the 30-days follow-up. This difference was confirmed by the log-rank test, which showed a statistically significant difference in survival distributions between subgroups (χ^2^ = 7.8, *p* < 0.05) (Figure 3A). To assess whether the adverse outcomes observed in influenza patients with elevated hsTnI were driven by specific components of the composite endpoint, we conducted subanalyses of the individual adverse events. In this study we observed higher rates of elevated hsTnI among patients who died, required mechanical ventilation, or were admitted to the ICU (Figure 3B). Consistently, Kaplan–Meier-transformed survival analyses revealed a significantly increased risk (*p* < 0.0001) for all-cause mortality, mechanical ventilation, and admission to the intensive care unit (Supplemental Figures S1–S3). To determine whether this critically elevated risk of adverse events in patients with positive hsTnI was influenced by key patient characteristics, we performed a multivariable regression analysis assessing the risk of the composite endpoint. In the model, hsTnI emerged as the only significant predictor, even after adjusting for age, sex, influenza type, cardiovascular risk factors, and comorbidities (Supplemental Table S1).

### 3.4. Integrating Troponin I into Machine Learning Models Improves Prognostic Accuracy in Influenza Patients

To improve risk stratification of influenza patients, we aimed to predict the composite endpoint by integrating key laboratory parameters, including hsTnI. Therefore, we compared the performance of various machine learning algorithms, including extreme gradient boosting (XGBoost), least absolute shrinkage and selection operator (LASSO), elastic net, forward selection, boosted tree, decision tree, neural boosted networks, support vector machine, and a classical nominal logistic regression model (Supplemental Figure S4). Among the cross-validated models, XGBoost demonstrated the highest predictive accuracy, with a significantly (*p* < 0.0001) lower median absolute error (MAE) compared to other models (Supplemental Figure S4). Subsequently, XGBoost was selected for further prediction of the composite endpoint in influenza patients.

**Figure 4:**
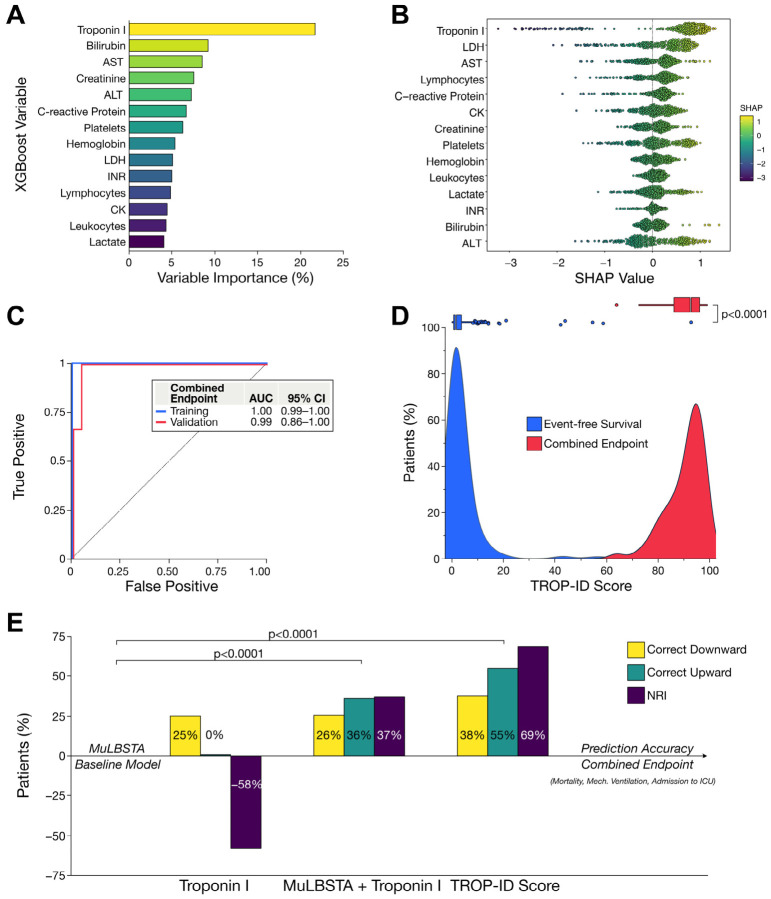
Prognostic modeling of adverse outcomes in influenza using hsTnI-based machine learning. (**A**) Feature importance ranking in the XGBoost model, identifying hsTnI as the strongest predictor of the composite endpoint. (**B**) SHapley Additive exPlanations (SHAP) plot visualizing the impact of individual predictors. Positive SHAP values indicate an increased likelihood for the composite endpoint, while negative SAHP values indicate lower likelihood of the outcome, with hsTnI showing the strongest association with adverse outcomes. (**C**) Receiver operating characteristic (ROC) analysis of the XGBoost algorithm to predict the composite endpoint by integration of laboratory data, including hsTnI. The supervised cross-validated machine learning model demonstrated a high diagnostic accuracy based on area under the curve (AUC). The model was trained and validated using a 10-fold cross-validation loop. ROC curves demonstrating excellent predictive performance of the XGBoost model in both training (AUC = 1.00) and test (AUC = 0.99) cohorts. (**D**) Based on the cross-validated model, patients were stratified according to the transformed likelihood for adverse outcomes utilizing the TROP-ID risk estimate, demonstrating significantly (*p* < 0.0001) higher scores in those with adverse outcomes. (**E**) Net reclassification improvement (NRI) comparing the validated MuLBSTA score, isolated hsTnI, the combined MuLBSTA + hsTnI model, and the TROP-ID model. The TROP-ID model showed the highest accuracy (accuracy = 0.826; PPV = 0.652; NPV = 1.00) and outperformed traditional risk models in predicting adverse outcomes in influenza patients.

Of note, among all laboratory parameters, high-sensitivity troponin I (hsTnI) emerged as the most important predictor of the composite endpoint (Figure 4A). Furthermore, a detailed comparison of individual features revealed distinct relationships between their values and the occurrence of adverse events (Figure 4B). Notably, hsTnI demonstrated the strongest association with the primary outcome, as indicated by its prominent SHapley Additive exPlanations (SHAP) values (Figure 4B). The XGBoost model demonstrated excellent predictive performance in identifying patients at risk for the composite endpoint in both the training cohort (ROC AUC = 1.00; 95% CI 0.99–1.00) and the test cohort (ROC AUC = 0.99; 95% CI 0.86–1.00) (Figure 4C). In the overall cohort, the model achieved a sensitivity of 100% and a specificity of 98.6%, underscoring its high accuracy in detecting influenza patients at elevated risk for poor outcomes. Furthermore, the transformed risk likelihood of the model, the Troponin I in Influenza Deterioration (TROP-ID) score, demonstrated a high level of accuracy in distinguishing patients at risk of disease progression from those without adverse events. Thus, the TROP-ID score significantly (*p* < 0.0001) differentiated between patients who experienced adverse events and those with event-free survival (Figure 4D). In order to assess the clinical relevance of the predictive score, we compared the accuracy of the TROP-ID risk estimate in stratifying patients at risk (Figure 4E). Therefore, net reclassification improvement (NRI) was calculated to assess the added value of clinical predictors for the 30-day composite endpoint. Risk predictions were derived from both Cox proportional hazards models and XGBoost models, and patients were stratified into low-, intermediate-, and high-risk groups based on tertiles of the predicted risk scores. NRI was then computed by evaluating appropriate upward or downward reclassification of events and non-events between models. At first, the MuLBSTA score, a clinical tool developed to predict adverse events in patients with viral pneumonia served as baseline model. The complex and validated model focuses on radiographic, laboratory, and patient-related characteristics and could correctly stratify 55.9% of patients (accuracy 0.559; positive predictive value (PPV) = 0.353; negative predictive value (NPV) = 0.765). We then compared the performance of isolated hsTnI assessment and noticed a drop in predictive accuracy (NRI = −0.58) when compared to baseline (Figure 4E). However, the addition of hsTnI to the baseline MuLBSTA model led to a significant (*p* < 0.0001) improvement stratification of influenza patients (NRI = 0.37) (Figure 4E). Ultimately, prediction of adverse events by the TROP-ID risk estimate significantly (*p* < 0.0001) outperformed the MuLBSTA model (NRI = 0.69) and thus, exhibited the highest diagnostic accuracy in correctly identifying influenza patients at risk for poor outcomes (Figure 4E). The simplified, tertile-based classification achieved an accuracy of 0.826 with a PPV of 0.652 and NPV of 1.

## 4. Discussion

The major findings of the present study are: (1) Increased high-sensitivity troponin I (hsTnI) is associated with poor prognosis in influenza patients. (2) Positive hsTnI is associated with all-cause mortality, the need for mechanical ventilation, and ICU admission. (3) Integrating laboratory data, including hsTnI, into machine learning models leads to high diagnostic accuracy in identifying influenza patients at high risk for adverse outcomes.

Our findings indicate that the assessment of hsTnI in combination with machine learning represents a valuable approach for stratifying the individual risk of adverse events in patients with influenza. Machine learning models incorporating hsTnI outperformed conventional risk stratification tools and validated clinical scores and may therefore contribute to improved patient management and clinical outcomes during seasonal influenza outbreaks. The current findings support existing evidence highlighting the significant morbidity and mortality associated with seasonal influenza. Elevated hsTnI, a marker of myocardial injury, may contribute to ventricular dysfunction in influenza patients, as demonstrated in previous research [14,27]. Consistent with prior studies suggesting an increased incidence of arrhythmias and deterioration of regional or global left ventricular function accompanied by increased mortality during viral infections, we found that hsTnI was inversely associated with left ventricular capacity [28,29,30].

Our findings are consistent with previous studies demonstrating the prognostic relevance of myocardial injury during influenza infection. A previous study reported that myocardial injury was strongly associated with death, ICU admission, and mechanical ventilation in hospitalized influenza patients [31], while another identified hsTnT as an independent predictor of acute cardiac events [32]. Similarly, the GEMINI initiative recently showed that troponin elevation is as common in patients hospitalized for influenza as in those with COVID-19, despite a relatively low rate of clinically recognized cardiac diagnoses [33]. Systemic inflammation has long been recognized as a key driver of the development and progression of atherosclerosis [34]. While previous studies have reported an increased incidence of acute coronary syndromes (ACSs) at the onset of influenza infections, in this study, the need for coronary angiography or percutaneous coronary intervention (PCI) did not impact prognosis during the follow-up period [35]. Additionally, exacerbation of preexisting coronary artery disease (CAD) by inflammation such as influenza may lead to plaque rupture and terminate in ACS, often accompanied by drastic consequences [36]. In this study, however, preexisting diagnosis of coronary artery disease (CAD) was not associated with an increased risk during follow-up, whereas isolated elevation of hsTnI was associated with adverse events.

Age, sex, serum creatinine, and C-reactive protein (CRP) were therefore included as covariates in multivariable analyses, as they represent well-established determinants of both influenza severity and cardiovascular risk. Older age and male sex are consistently associated with increased mortality, ICU admission, and myocardial injury during influenza infection [7,37]. Renal dysfunction, reflected by elevated creatinine, is strongly associated with poor prognosis and may significantly influence troponin concentrations independent of acute myocardial injury, particularly in patients with CAD [38,39]. Similarly, CRP reflects systemic inflammation and infection severity, both of which contribute to endothelial dysfunction, plaque destabilization, and influenza-associated myocardial injury [40]. Adjusting for these variables allowed a more robust assessment of the independent prognostic value of hsTnI. Accordingly, patients with elevated hsTnI showed a fourfold higher risk of the composite endpoint and a markedly increased 30-day cumulative hazard (41.4% vs. 10.4%), supporting hsTnI as an independent predictor of adverse short-term outcomes beyond established clinical risk factors. Moreover, in line with previous studies, a higher burden of baseline cardiovascular comorbidities in patients with elevated hsTnI suggests that troponin elevation may partly reflect preexisting cardiovascular vulnerability in addition to acute myocardial injury during influenza infection [31,41]. However, even after adjustment for these comorbidities, hsTnI remained the strongest independent predictor of adverse outcomes, indicating prognostic value beyond baseline cardiovascular risk alone. This supports the concept that hsTnI reflects clinically relevant myocardial stress triggered by influenza rather than merely chronic cardiovascular disease [42,43]. Nonetheless, residual confounding of preexisting cardiovascular disease on the prognostic importance of hsTnI cannot be fully excluded due to the retrospective design and cardiovascularly enriched study population.

Elevated hsTnI may therefore indicate direct myocardial injury caused by viral infection, potentially leading to adverse outcomes [42]. In addition to direct viral invasion, mechanisms such as thromboinflammation, endothelial activation, microcirculatory imbalance, and increased vulnerability of atherosclerotic plaques may also contribute to complications in patients with influenza [35,44]. In this context, recent evidence suggest that the protective effects of influenza vaccination may extend beyond infection prevention alone and may involve immunomodulatory mechanisms promoting resolution of inflammation and potentially attenuating myocardial injury [45].

Moreover, late mortality in influenza patients may be primarily driven by bacterial superinfections and pneumonia, which can lead to a more severe disease course [46]. These factors are incorporated into the clinically validated MuLBSTA score, a tool to stratify individual risks for adverse events [47]. Strikingly, incorporating hsTnI into machine learning models in this study significantly outperformed the established risk score, thereby enhancing the identification of patients at risk for adverse outcomes. The superior performance of machine learning models incorporating hsTnI compared with the MuLBSTA score highlights the added value of cardiac biomarker integration for influenza risk stratification. While the MuLBSTA score, originally developed for viral pneumonia, demonstrated moderate prognostic performance, it mainly relies on clinical and demographic variables without directly reflecting myocardial injury [47]. Our findings suggest that hsTnI provides important complementary prognostic information, improving the identification of patients at increased risk for mortality, ICU admission, and mechanical ventilation. This further supports the concept that the clinical value of biomarkers in severe influenza requires better definition and highlights hsTnI as a promising tool for more precise risk stratification [48]. Further, our findings together with recent studies suggest that the integration of biomarkers such as hsTnI with clinically validated parameters and modern machine learning approaches may serve as a practical and effective tool for ICU triage, optimized resource allocation, and individualized patient management during seasonal influenza outbreaks [49].

Our study has several clinical implications: First, hsTnI predicts adverse outcomes in patients with seasonal influenza, and by integrating hsTnI into machine learning models, we achieved superior risk stratification compared to established clinical scores. These findings highlight the clinical utility of hsTnI as a readily available, cost-effective biomarker to assist in the early identification of influenza patients at high-risk. Second, the findings may further enable targeted monitoring and intervention to improve outcomes. Elevated hsTnI levels may support clinical decision-making for ICU admission, optimizing resource allocation during seasonal influenza peaks or pandemics. Conversely, negative hsTnI and low TROP-ID risk may be considered for early discharge or outpatient follow-up, improving hospital economy. Thus, routine assessment of hsTnI may improve diagnostic accuracy for stratifying influenza patients and ultimately enhance patient management and outcomes. Future prospective multicenter studies are needed to externally validate the TROP-ID score and confirm the prognostic value of hsTnI-guided machine learning models in broader influenza populations. In addition, protocolized serial troponin measurements may help distinguish acute from chronic myocardial injury and further improve ICU triage, discharge planning, and long-term cardiovascular follow-up.

### 4.1. Conclusions

Among hospitalized patients with seasonal influenza, myocardial injury identified by elevated high-sensitivity troponin I is strongly associated with poor short-term clinical outcomes, including mortality, ICU admission, and mechanical ventilation. hsTnI remained an independent predictor of adverse events even after adjustment for cardiovascular comorbidities and established clinical risk factors. Integration of hsTnI into machine learning-based prediction models significantly improved risk stratification beyond conventional clinical scores such as MuLBSTA. Routine hsTnI assessment may therefore represent a simple, cost-effective, and clinically valuable tool for early identification of high-risk influenza patients and may support improved individualized management strategies.

### 4.2. Limitations

This present study has certain limitations: The retrospective design and single-center setting may limit the generalizability of the findings. However, the focused assessment of hospitalized patients with seasonal influenza enabled a detailed clinical evaluation and robust follow-up. Despite a moderate sample size, the study captured a substantial number of adverse events, allowing for a robust stratification of patients by hsTnI. The cohort represents a cardiovascular-enriched population, as patients were admitted to a cardiology department, which may limit generalizability to unselected influenza populations. While external validation in independent cohorts is warranted to confirm the predictive performance of the machine learning models and the prognostic relevance of hsTnI, the current cross-validated findings provide a strong foundation for further investigation. The observed decline in hsTnI among patients with adverse outcomes may also reflect measurement timing relative to peak myocardial injury, rather than a protective effect. This finding should therefore be interpreted cautiously. Additionally, data on influenza vaccination status, antiviral treatment, and detailed disease severity markers (e.g., ARDS severity, lactate) were not consistently available and may confound the observed associations. Especially vaccination is known to reduce cardiovascular complications and mortality and may therefore have influenced both clinical outcomes and the interpretation of hsTnI elevation. However, whether vaccination directly affects troponin release during acute influenza infection remains insufficiently studied and requires dedicated prospective investigation. Nevertheless, the integration of a validated biomarker, hsTnI, into predictive modeling offers a potentially valuable tool for early risk stratification and improved clinical management of influenza patients.

## Figures and Tables

**Table 1:** Baseline characteristics of patient population.

	All	Troponin Positive	Troponin Negative	*p*-Value
	**(*n* = 277)**	**(*n* = 105; 37.9%)**	**(*n* = 172; 62.1%)**	
Female, n (%)	127 (45.9)	38 (36.2)	89 (51.7)	**0.012**
Age, years (median, IQR)	72 (60–80)	76 (66–83)	68.5 (56–78)	**<0.001**
**Cardiovascular risk factors**				
Arterial hypertension, n (%)	175 (63.2)	79 (75.2)	96 (55.8)	**0.001**
Hyperlipidemia, n (%)	72 (26)	41 (39.1)	31 (18)	**0.001**
Diabetes mellitus, n (%)	75 (27.1)	43 (41)	32 (18.6)	**0.001**
Current smoking, n (%)	52 (18.8)	20 (19.1)	32 (18.6)	0.927
Obesity, n (%)	58 (20.9)	19 (18.1)	39 (22.7)	0.364
Coronary artery disease, n (%)	97 (35)	56 (53.3)	41 (23.8)	**<0.001**
Percutaneous coronary intervention, n (%)	26 (9.4)	23 (21.9)	3 (1.7)	**<0.0001**
LVEF (%) (median, IQR)	55 (50–60)	55 (44–55)	60 (55–60)	**<0.001**
**Medication on admission**				
Statins, n (%)	96 (34.7)	49 (46.7)	47 (27.3)	**0.001**
Acetylsalicylic acid, n (%)	65 (23.5)	26 (24.8)	39 (22.7)	0.692
Clopidogrel, n (%)	12 (4.3)	6 (5.7)	6 (3.5)	0.390
Ticagrelor, n (%)	5 (1.8)	2 (1.9)	3 (1.7)	0.923
Prasugrel, n (%)	3 (1.8)	1 (1)	2 (1.2)	0.869
Oral anticoagulants, n (%)	51 (18.4)	30 (28.6)	21 (12.2)	**0.001**
Angiotensin-converting enzyme inhibitors, n (%)	90 (32.5)	42 (40)	48 (27.9)	**0.038**
Angiotensin II receptor antagonists, n (%)	47 (17)	23 (21.9)	24 (14)	0.091
Aldosterone antagonists, n (%)	32 (11.6)	20 (19.1)	12 (7)	**0.003**
Ca channel antagonists, n (%)	73 (26.4)	37 (35.2)	36 (20.9)	**0.009**
β-blockers, n (%)	135 (48.7)	71 (67.6)	64 (37.2)	**<0.0001**
Diuretics, n (%)	106 (38.3)	59 (56.2)	47 (27.3)	**<0.0001**
**Laboratory parameters**				
GFR-MDRD (ml/min/1.73 m^2^) (median, IQR)	68.5 (47.1–86.7)	70.8 (32.2–70.8)	74.4 (60.9–95.5)	**<0.0001**
C-reactive protein (mg/dL) (median, IQR)	2.9 (1.5–7.9)	5.6 (2.2–10.7)	2.3 (1.3–5.4)	**<0.0001**
Leukocytes (10^9^/L) (median, IQR)	7.1 (5.3–9.4)	7.7 (5.8–10)	6.6 (5.1–9.2)	**0.003**
Lymphocytes (10^6^/L) (median, IQR)	0.9 (0.6–1.4)	0.9 (0.5–1.4)	0.9 (0.6–1.4)	0.259
Platelets (10^9^/L) (median, IQR)	178 (142–225.8)	174.5 (137.3–220)	182 (146–234.8)	0.192
Hemoglobin (g/dL) (median, IQR)	13.1 (11.9–14.1)	12.7 (11–14)	13.3 (12.2–14.2)	**0.007**
Lactate (mmol/L) (median, IQR)	1.5 (1.1–2)	1.7 (1.2–2.5)	1.3 (1–1.7)	**<0.0001**
AST (U/L) (median, IQR)	40 (25–86.5)	43 (28–128)	32 (22.5–61)	**0.003**
ALT (U/L) (median, IQR)	23 (16–36)	24 (18–46)	22 (16–35)	**0.044**
LDH (U/L) (median, IQR)	240 (194.8–303.5)	291 (235–387.5)	212 (185.5–265.5)	**<0.0001**
Total cholesterol (mg/mL) (median, IQR)	134 (114–185)	125.5 (113–181)	204 (106.5–235.5)	0.180
Creatine Kinase (U/L) (median, IQR)	142 (76–373)	283 (93.3–764.5)	119.5 (63.8–237)	**<0.0001**
NT-proBNP (ng/L) (median, IQR)	2204 (407–7636)	6734 (1272–14938)	393 (93–2210)	**<0.0001**
**Disease severity**				
Mild ARDS, n (%)	84 (30.3)	47 (44.1)	37 (21.5)	
Moderate ARDS, n (%)	14 (5.1)	12 (11.4)	2 (1.2)	
Severe ARDS, n (%)	18 (6.5)	13 (12.4)	5 (2.9)	
First Horowitz index (mmHg) (median, IQR)	153 (103–221)	148 (102–212)	178 (120–425)	0.288
MuLBSTA score (median, IQR)	6 (4–8)	8 (4–9)	4 (4–8)	**<0.0001**
Viral coinfection, n (%)	9 (3.3)	6 (5.7)	3 (1.7)	0.076
Bacterial coinfection, n (%)	19 (6.9)	13 (12.4)	6 (3.5)	**0.005**
**Follow-Up**				
Death, n (%)	33 (11.9)	24 (22.9)	9 (5.2)	**<0.0001**
Mechanical Ventilation, n (%)	30 (10.8)	25 (23.8)	5 (2.9)	**<0.0001**
Admission to ICU, n (%)	50 (18.1)	40 (38.1)	10 (5.8)	**<0.0001**

Significant values (*p* < 0.05) are highlighted in bold.

## Data Availability

The data that support the findings of this study are available on reasonable request from the corresponding author.

## Supplementary Materials

The following supporting information can be downloaded at: https://www.mdpi.com/article/10.3390/jcm15124509/s1. Table S1. Multivariable regression analysis of predictors for the composite endpoint in influenza patients. Figure S1. Elevated hsTnI levels are associated with increased all-cause mortality in influenza patients. Figure S2. Association between hsTnI elevation and need for mechanical ventilation. Figure S3. Elevated hsTnI predicts ICU admission in patients with seasonal influenza. Figure S4. Comparison of machine learning models for prediction of adverse outcomes in influenza.
