# Supplementary material for: Routine Troponin I Assessment Enhances Risk Stratification in Hospitalized Patients with Seasonal Influenza
Source: J Clin Med. 2026 Jun 10;15(12):4509. doi: 10.3390/jcm15124509 (PMC13301208; doi:10.3390/jcm15124509)
Supplement: Supplementary file 1 [file jcm-15-04509-s001.zip › jcm-4289258-supplementary.pdf]

Routine Troponin I Assessment Enhances Risk Stratification in Hospitalized Patients with Seasonal Influenza

Tobias Harm [1], Johannes Gernert [1], Monika Zdanyte [1], Lars Schöllmann [2], Karin Anne Lydia Müller [1], Meinrad Paul Gawaz [1], Dominik Rath [1] and Simon Greulich [1,*]

[1] Department of Cardiology and Angiology, University Hospital Tübingen, Eberhard Karls University Tübingen, 72076 Tübingen, Germany
[2] Department of Cardiology, Medius Clinic Ostfildern-Ruit, 73760 Ostfildern, Germany
* Correspondence: simon.greulich@med.uni-tuebingen.de; Tel.: +49-(0)-7071-29-83688

Supplemental Tables and Figures

Supplemental Table S1. Multivariable regression analysis of predictors for the composite endpoint in influenza patients.

Multivariable Regression Analysis (p<0.0001)			
**Variable**	**HR**	**95% CI**	**p-Value**
Troponin I+	6.71	3.13-14.4	**<0.0001**
Age	1.03	1.00-1.06	0.046
Female	0.92	0.46-1.85	0.820
Influenza A/B	1.48	0.73-3.02	0.277
Coronary Artery Disease	0.40	0.18-0.88	0.022
Arterial Hypertension	1.42	0.60-3.34	0.420
Smoking	1.93	0.80-4.65	0.141
Obesity	0.42	0.15-1.14	0.087
Diabetes Mellitus	0.85	0.39-1.86	0.685
Chronic Kidney Disease	2.64	1.10-6.31	0.029
Active Malignancy	2.57	1.13-5.83	0.025

95% CI, 95% Confidence Interval; OR, Odds Ratio.

Nominal logistic regression model identifying independent predictors of adverse outcomes (death, ICU admission, or mechanical ventilation) within 30 days. High-sensitivity troponin I (hsTnI) was the only independent predictor after adjusting key patient characteristics.

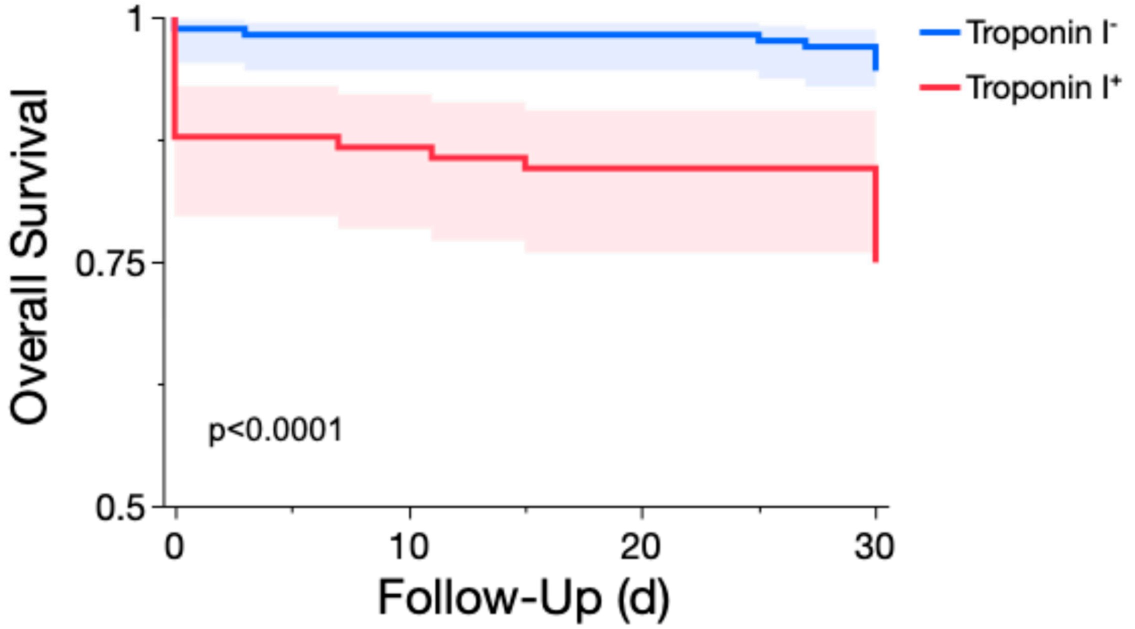

Supplemental Figure S1. Elevated hsTnI levels are associated with increased all-cause mortality in influenza patients.

Kaplan–Meier survival analysis showing significantly reduced 30-day survival in patients with elevated high-sensitivity troponin I (hsTnI) compared to those with normal hsTnI levels (p<0.0001).

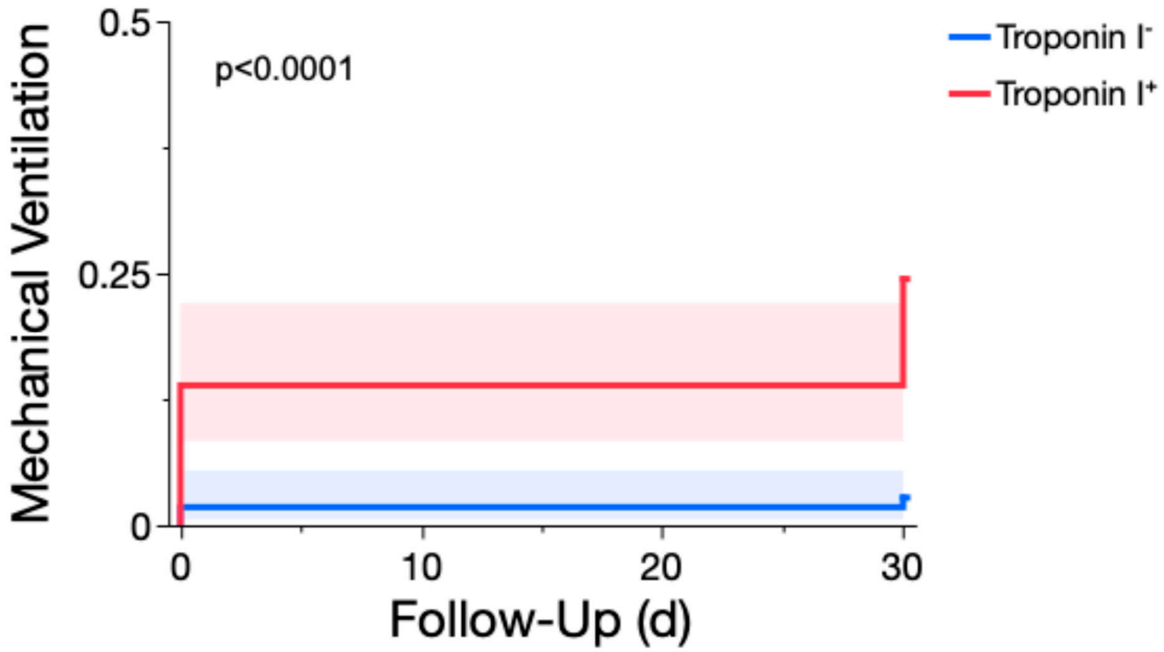

Supplemental Figure S2. Association between hsTnI elevation and need for mechanical ventilation.

Kaplan–Meier analysis reveals a significantly higher risk of mechanical ventilation in influenza patients with elevated hsTnI levels during the 30-day follow-up ($p < 0.0001$).

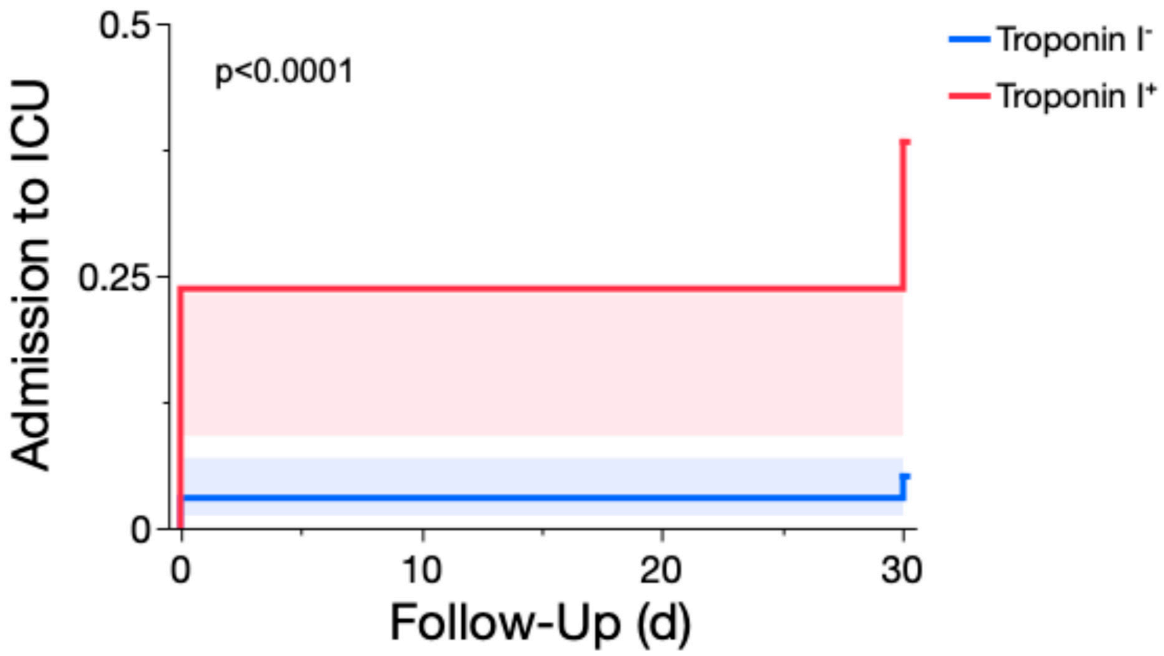

Supplemental Figure S3. Elevated hsTnI predicts ICU admission in patients with seasonal influenza.

Kaplan–Meier survival curves demonstrate a significantly increased rate of ICU admissions among patients with elevated hsTnI levels ($p < 0.0001$).

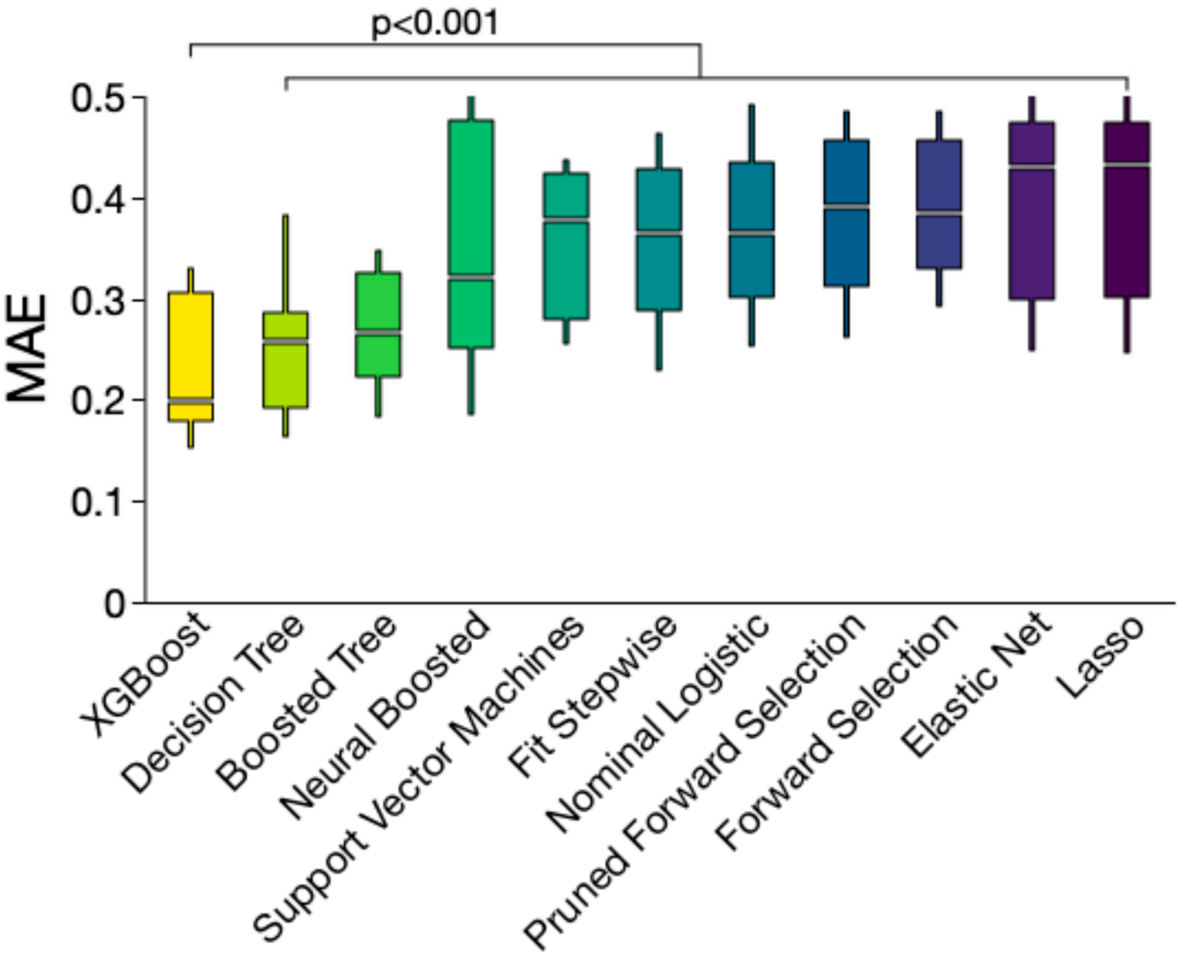

Supplemental Figure S4. Comparison of machine learning models for prediction of adverse outcomes in influenza.

Median absolute error (MAE) of various machine learning models trained to predict the composite endpoint in influenza patients. XGBoost demonstrated the lowest error and highest accuracy ($p < 0.0001$), outperforming classical and other ML-based approaches.
